# Phosphoproteomic Analysis Reveals a Different Proteomic Profile in Pediatric Patients With T-Cell Lymphoblastic Lymphoma or T-Cell Acute Lymphoblastic Leukemia

**DOI:** 10.3389/fonc.2022.913487

**Published:** 2022-07-08

**Authors:** Giulia Veltri, Federica Lovisa, Giuliana Cortese, Marta Pillon, Elisa Carraro, Simone Cesaro, Massimo Provenzi, Salvatore Buffardi, Samuela Francescato, Alessandra Biffi, Barbara Buldini, Valentino Conter, Valentina Serafin, Lara Mussolin

**Affiliations:** ^1^Maternal and Child Health Department, University of Padova, Padova, Italy; ^2^Oncohematology, Stem Cell Transplant and Gene Therapy Research Area, Istituto di Ricerca Pediatrica Città della Speranza, Padova, Italy; ^3^Department of Statistical Sciences, University of Padova, Padova, Italy; ^4^Clinic of Pediatric Oncohematology, University Hospital of Padova, Padova, Italy; ^5^Pediatric Hematology-Oncology, Woman and Child Hospital, Azienda Ospedaliera Universitaria Integrata, Verona, Italy; ^6^Pediatric Hematology and Oncology Unit, Papa Giovanni XXIII Hospital, Bergamo, Italy; ^7^Pediatric Haemato-Oncology Department, Santobono-Pausilipon Children’s Hospital, Napoli, Italy; ^8^Pediatric Hematology Oncology Unit, University of Milano-Bicocca, Monza e Brianza per il Bambino e la sua Mamma (MBBM) Foundation, Azienda Socio Sanitaria Territoriale (ASST) Monza, Monza, Italy; ^9^Department of Surgery Oncology and Gastroenterology, Oncology and Immunology Section, University of Padova, Padova, Italy

**Keywords:** T-LBL, T-ALL, phosphoproteomics analysis, AKT/mTOR, JAK/STAT (janus kinase/signal transducer and activator of transcription)

## Abstract

T-cell lymphoblastic lymphoma (T-LBL) and lymphoblastic leukemia (T-ALL) arise from the transformation of precursor T-cells sharing common morphological and immunophenotypic features. Despite this, T-LBL and T-ALL show different genomic/transcriptomic profiles and whether they represent two distinct disease entities or variant manifestations of the same disease is still a matter of debate. In this work, we performed a Reverse Phase Protein Array study on T-LBL and T-ALL samples and demonstrated that they are characterized by a different phosphoproteomic profile. Indeed, T-LBLs showed the hyperactivation of FAK/ERK1/2 and AKT/mTOR pathways, whereas JAK/STAT pathway was significantly hyperphosphorylated in T-ALLs. Moreover, since the only criteria for discriminating T-LBL from T-ALL is blasts’ infiltration below 25% in the bone marrow and lymphoma patients can present with a percentage of blasts close to this cut-off, a biomarker that could help distinguishing the two diseases would be of great help for the clinical diagnosis and treatment decision. Pursuing this aim, we identified a proteomic signature of six proteins whose expression/activation was able to discriminate stage IV T-LBL from T-ALL. Moreover, we demonstrated that AKT hyperphosphorylation alone was able to distinguish stage IV T-LBL from both T-ALL and stage III T-LBL. Concluding, these data demonstrate that T-ALL and T-LBL bear different phosphoproteomic profiles, further sustaining the hypothesis of the two disease as different entities and paving the way for the identification of new biomarkers able to distinguish stage IV T-LBL from T-ALL disease, so far based only on BM involvement criteria.

## Introduction

T-cell lymphoblastic leukemia (T-ALL) and lymphoblastic lymphoma (T-LBL) arise from the transformation of immature precursor T-cells sharing common morphological and immunophenotypic features (1). T-ALL comprises 10-15% of pediatric ALL cases (2), whereas T-LBL accounts for 20-30% of all non-Hodgkin lymphomas in children (3). Despite the World Health Organization (WHO) and the International Lymphoma Study Group classify T-ALL and T-LBL together as T-lymphoblastic leukemia/lymphoma (4, 5), there is still an ongoing discussion on whether they represent distinct disease entities or variant manifestations of the same disease (6). Many clinical trials use bone marrow blast infiltration as the sole criterion to distinguish T-ALL from T-LBL. By convention, T-LBL is diagnosed when the neoplasm presents with < 25% T-lymphoblasts in the bone marrow (BM) and T-ALL if this percentage is higher (5, 7).

Over the last decade, the genetic profiles of these malignancies have been investigated and there are now clear indications from (epi)genetic and gene expression studies that T-ALL and T-LBL may be biologically different in certain aspects (8–11). T-LBL cells were shown to express increased levels of S1P1 and ICAM1 compared with T-ALL and these features were proved to account for the lack of T-LBL cells dissemination (8). In line with those findings, a previous study by our research group identified a subset of differentially expressed genes in T-LBL compared to T-ALL, which included genes involved in chemotactic responses and angiogenesis and possibly playing a role in tumor cell localization. In addition, genome-wide copy number alteration analysis revealed that, although most aberrations were found in both entities, several were selectively identified in T-LBL or T-ALL (9). T-LBL-specific gene variants were also identified by whole-exome sequencing, further supporting the concept of distinct molecular mechanisms for T-LBL and T-ALL disease onset and clinical behavior (10). More recently, DNA methylation profiles were also investigated and several differentially methylated CpG islands were identified between T-LBL and T-ALL clinical samples, even though the functional relevance of these methylation disparities still remains to be elucidated (11).

To the best of our knowledge, no data are available regarding comparison of T-ALL and T-LBL phospho-proteomic profiles. In the present work, we applied the Reverse Phase Protein Array (RPPA) technology on a series of pediatric T-ALL and T-LBL samples, to identify signaling pathways that could be differentially activated in the two diseases, in pursuit of additional classification criteria, potentially useful to define the most appropriate treatment approach.

## Methods

### Patients

Diagnostic tumor specimens from 22 T-LBL and bone marrow (BM) aspirates at diagnosis from 57 T-ALL were analyzed in this study. Samples were collected at the Pediatric Oncohematology Laboratory of the University of Padua (Padua, Italy) between 1999 and 2017. According to Helsinki’s Declaration, all patients’ samples were obtained after obtaining informed consent.

T-LBL patients were enrolled in AIEOP LNH-97 (12) or EuroLB-02 (13) treatment protocols. The diagnosis of T-LBL was established according to the WHO guidelines (5) and centrally reviewed in all the cases. The median age was 10.1 years (range 2.2–18.5). All the patients were diagnosed with stage III or IV disease, according to the St Jude’s classification (14). Mutational analysis of *PTEN* and *PIK3CA* hot-spot exons was performed as previously reported (15, 16)

T-ALL pediatric patients were enrolled in the AIEOP-BFM ALL2000/ALL2009 protocols (17, 18). The median age was 8.7 years (range 1.8–17.83). The classification of the T-ALL subtypes followed European Group for the Immunologic Characterization of Leukemias (EGIL) and World Health Organization (WHO) 2016 criteria (5, 19). The study was approved by the ethical committee board of the Comitato Etico per la Sperimentazione Clinica della Provincia di Padova and the Italian Association of Pediatric Onco-Hematology (AIEOP). The main clinical characteristics of the study cohort are reported in **Table 1**.

**Table 1:** Main clinical characteristic of T-ALL and T-LBL patients included in the study.

T-ALL patients (N= 57)		T-LBL patients (n=22)	
**Gender (n, %)**		**Gender (n, %)**	
Male	44 (77%)	Male	18 (82%)
Female	13 (23%)	Female	4 (18%)
**Age, years (n,%)**		**Age, years (n, %)**	
> 8.7	27 (47%)	> 10.1	11 (50%)
≤ 8.7	30 (53%)	≤ 10.1	11 (50%)
**Immunophenotype (n, %)**		**Stage (n, %)**	
Early T (T-I and T-II)	6 (12%)	III	13* (60%)
Intermediate T (T-III or cortical)	43 (75%)	IV	9^#^ (40%)
Mature T (T-IV)	6 (10%)	**BM involvement (n, %)**	
NA	2 (3%)	yes	7 (32%)
**Response to Prednisone (n, %)**		no	15 (68%)
Good	28 (50%)	**CNS involvement (n, %)**	
Poor	20 (35%)	yes	1 (5%)
NA	9 (15%)	no	21 (95%)
**Follow-up (n, %)**		**Follow-up (n, %)**	
Complete remission	NA	Complete remission	15 (68%)
Relapse	4 (7%)	Relapse	3 (13.5%)
		Progression	3 (13.5%)
		Resistance	1 (5%)

BM, bone marrow; CNS, central nervous system; NA, not available.

*5/13 relapsed/resistant.

^#^2/9 relapsed/resistant.

### Reverse Phase Protein Array

RPPA technique was performed as previously reported (20, 21). Shortly, mononuclear cells from T-ALL BM samples were isolated using the Ficoll-Hypaque technique (Pharmacia, Sigma-Aldrich, St. Louis, MO). T-LBL tumor samples were either lymph node biopsies (18/22) or pleural effusions (4/22). Mononuclear cells from T-ALL BM samples and dried cell pellets from T-LBL pleural effusions were lysed in T-PER™ Tissue Protein Extraction Reagent (Thermo Scientific™, Waltham, MA) plus protease and phosphatase inhibitors (Sigma-Aldrich, St. Louis, MO). The same lysis buffer with an additional disintegration step by a manual pestle has been used for protein extractions from lymph node biopsies. All lysates were printed in a 4-point dilution curve in quadruplicate on nitrocellulose-coated glass slides (ONCYTE^®^ Nitrocellulose Film Slides, Grace BioLabs, Bend, OR), using the 2470 Arrayer (AushonBioSystems, Billerica, MA). The slides were automatically stained with 53 primary antibodies already validated by Western Blot (WB) for single band specificity (**Supplementary Table S1**), by using an automated slide stainer (DakoAutostainer Plus, Dako-Cytomation, Santa Clara, CA). Signal amplification was performed employing the TSA PLUS BIOTIN system (AKOYA Biosciences, Marlborough, MA) and then the signal was revealed using diaminobenzidine/hydrogen peroxide (Dako-Cytomation, Santa Clara, CA) as a chromogen substrate after 5 minutes incubation. Microvigene software (VigeneTech Inc, Carlisle, MA) was used to extract absolute numeric intensity protein values from the array TIF images of each antibody and total protein.

### Western Blotting

SDS–polyacrylamide gel electrophoresis was performed using 4–20% Criterion TGX Stain-Free Protein Gel (Bio-Rad, Hercules, CA), and proteins were transferred to poly-vinylidene difluoride (PVDF) Immobilon-p membrane (Merck-Millipore, Billerica, MA) by wet/tank Bio-Rad blotting system (Bio-Rad). Membranes were blocked in 2% I-Block blocking buffer (Thermo Fisher Scientific) and incubated overnight at 4°C with primary antibodies. After two washes with PBS-T, blots were incubated with the HRP-conjugated secondary antibody (1:25000, PerkinElmer, Waltham, MA). Bands were detected using the iBright imaging system (Thermo Fisher Scientific). The following primary antibodies were used: AKT S473 (abcam, ab78403), AKT TOT (R&D, MAB1775), S6 RB S235/236 (Cell Signaling Technology, #2211) and GAPDH (Genetex, GTX 8627408).

### Statistical Analyses

To identify statistically significant differentially activated or expressed proteins between T-LBL and T-ALL patients, we applied the nonparametric Wilcoxon test. Multiplicity corrections were conducted following the false discovery rate (FDR) method (Benjamini-Hochberg procedure). Proteins were considered significantly differentially expressed/activated for a result with a corrected FDR ≤ 0.05. Hierarchical clustering has been conducted by using a multiple testing procedure to control the family-wise error rate (inheritance procedure) (22). The differential activation status between T-LBL and T-ALL patients was globally evaluated for all considered proteins, through a locally most powerful test (Global Test) (23). The classification performance based on a set of proteins for prediction of stage IV T-LBL was studied by a Breiman’s random forest algorithm for classification, with a cross-validation fold of 5 (24). These analyses were performed with the statistical software R (www.r-project.org).

## Results

**Figure 1:**
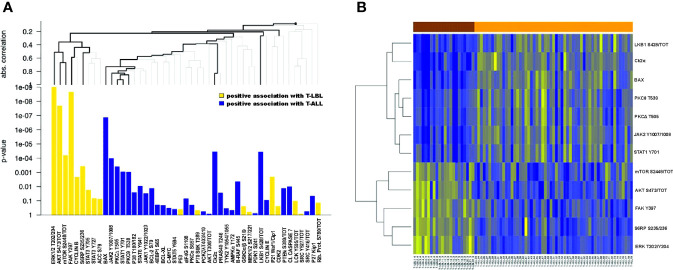
Differentially expressed proteins between T-LBL and T-ALL samples. **(A)** Hierarchical clustering graph (HCG) of all proteins, where black bold lines highlight those proteins being most significantly associated with T-LBL or T-ALL. Proteins are ordered by following the HCG, where the clustering method is the average linkage and the distance measure used for the graph is the absolute correlation distance. Positive association with T-LBL (or T-ALL) refers to hyperactivated proteins in T-LBL (or in T-ALL). **(B)** Heatmap of the ten most differentially expressed proteins between T-LBL and T-ALL samples.

From RPPA analysis we identified 24/47 proteins (51%) significantly differentially expressed between T-ALL and T-LBL patients, with a corrected FDR ≤ 0.05 (**Supplementary Figure S1** and **Supplementary Table S2**). This result was further investigated by a hierarchical clustering tree analysis, to also account for a possible correlation between proteins and simultaneously correcting for multiple testing. From this procedure, we identified a subgroup of 5 proteins with a significantly positive association with T-LBL and a subgroup of 7 proteins positively associated with T-ALL (**Figure 1A**, bold lines). **Figure 1B** shows these twelve most differentially expressed proteins between T-LBL (brown) and T-ALL (orange) patients. We then reconstructed the activation status of specific pathways linked to the most differentially expressed proteins through a locally most powerful test. Interestingly, the FAK/ERK1/2 pathway, which is known to be crucial for tumor growth, angiogenesis and vascular permeability in solid tumors (25, 26), was among the most significantly activated pathways in T-LBL compared to T-ALL patients (p<0.001). In line with this finding, gene set enrichment analysis (GSEA) using the MSigDB geneset Hallmark and Canonical pathways C2, KEGG subset (http://www.broad.mit.edu/gsea/) on our previously published gene expression (GEP) dataset (9) showed a positive enrichment for genes involved in angiogenesis and focal adhesion in T-LBL compared to T-ALL samples (**Supplementary Figures S2A, B**). Similarly, the AKT/mTOR pathway resulted significantly hyperactivated in T-LBL compared to T-ALL (p<0.001).

Conversely, a higher phosphorylation level of the JAK1/2-STATs pathway, except for STAT3 Y705 and S727, characterized T-ALL compared to T-LBL (p=0.002). In line with this, gene sets associated with IL6-JAK-STAT3 signaling were significantly upregulated in T-LBL compared to T-ALL patients (**Supplementary Figure S2C**)

Finally, proteins belonging to cell cycle, such as Cyclin B and p21, resulted differentially regulated between the two groups (p=0.002) (**Supplementary Figure S1** and **Supplementary Table S3**). Taken together, these results suggest that T-ALL and T-LBL in pediatric age are characterized by a different phosphoproteomic profile.

**Figure 2:**
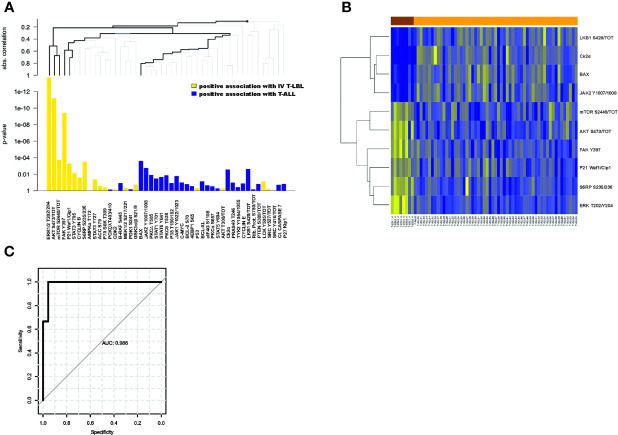
Differentially expressed proteins between stage IV T-LBL and T-ALL samples. **(A)** Hierarchical clustering graph (HCG) of all proteins, where black bold lines highlight those proteins being significantly most strongly associated with stage IV T-LBL or T-ALL. Positive association with stage IV T-LBL (or T-ALL) refers to hyperactivated proteins in stage IV T-LBL (or in T-ALL). **(B)** Heatmap of the ten most differentially expressed proteins between stage IV T-LBL and T-ALL samples. **(C)** ROC curve of the predicted probabilities of being classified as a T-ALL or stage IV T-LBL for data in the testing set, together with the estimated area under the ROC curve (AUC).

Another important question to be addressed was about T-LBL patients classified as stage IV. This clinical group, despite being diagnosed as T-LBL, can be characterized by a percentage of blasts in the BM close to the cut-off of 25% used to define T-ALL. Thus, an early biomarker that can successfully identify these patients at diagnosis would be of great help in the clinical decision. In light of this, we performed a global analysis of all the proteins comparing the phosphorylation profile of stage IV T-LBL samples with both T-ALL and stage III T-LBL tumors. Interestingly, we found that 17/47 proteins (36%) were significantly differentially expressed between stage IV T-LBL and T-ALL (**Supplementary Table S4**), with seven proteins positively associated with stage IV T-LBL compared to T-ALL, whereas ten proteins were positively associated with T-ALL (**Figure 2A**). **Figure 2B** shows the ten most differentially expressed proteins between stage IV T-LBL (brown) and T-ALL (orange), which included proteins belonging to the JAK/STATs, AKT/mTOR, FAK/ERK1/2 and cell cycle pathways. When focusing on GEP data from stage IV T-LBL and T-ALL samples (9), gene sets associated with angiogenesis, focal adhesion and IL6-JAK-STAT3 signaling resulted significantly upregulated in stage IV T-LBL compared to T-ALL patients (**Supplementary Figures S2D-F**).

**Figure 3:**
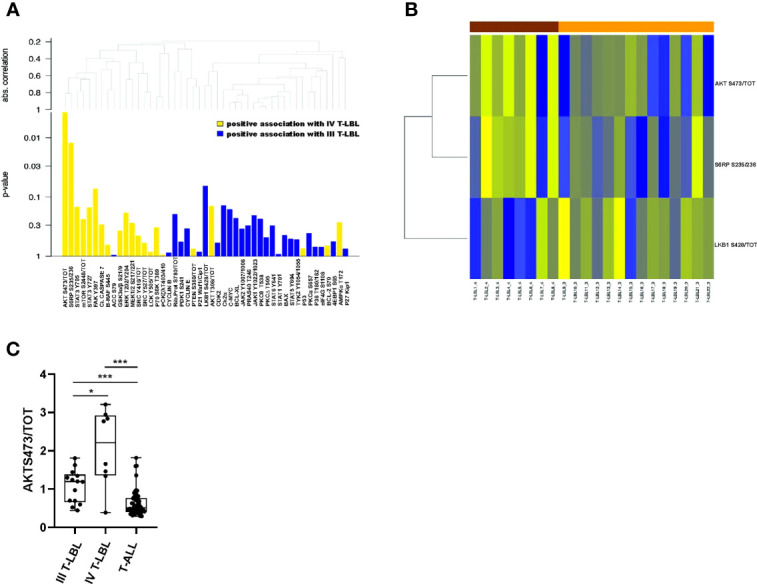
Differentially expressed proteins between stage IV and stage III T-LBL samples. **(A)** Hierarchical clustering graph (HCG) of all proteins, and a set of proteins being positively associated with stage IV or stage III T-LBL, with different intensities (height of columns is equal to p-values of single tests). Positive association with stage IV T-LBL (or T-ALL) refers to hyperactivated proteins in stage IV T-LBL (or in T-ALL). **(B)** Heatmap of the three significantly differentially expressed proteins between stage IV and stage III T-LBL samples (based on non-corrected p-values from single tests). **(C)** AKT S473/tot phosphorylation levels measured by RPPA analysis were significantly upregulated in stage IV T-LBL samples (n=8) compared to both T-ALL (n=57) and stage III T-LBL (n=14).*p < 0.05; ***p < 0.001.

Within this group, the classification tree highlighted six proteins (bold lines), classified in three groups: ERK1/2 T202/Y204, AKT S473/tot, mTOR S2448/tot; FAK Y397, P21; BAX. Thus, only for these six proteins, we investigated their global performance in correctly classifying stage IV T-LBL vs T-ALL. To this aim, we split the data into a 60% training set and a 40% test set, we then trained a random forest classification model with 5-fold cross-validation. Finally, based on the resulting trained model, we predicted the probability of being classified as T-ALL or stage IV T-LBL on the test set, plotting a ROC curve of these predictions (**Figure 2C**). The area under the ROC curve (AUC) revealed that, taken together, ERK1/2 T202/Y204, AKT S473/tot, mTOR S2448/tot, FAK Y397, P21 and BAX provide a very high prediction accuracy in classifying patients as stage IV T-LBL or T-ALL (AUC = 0.986; p = 0.0043, CI 95%: 0.945-1), with a high sensitivity (99.9%) and specificity (95.6%) selected on the optimal cut-off point provided by the Youden Index. When comparing stage III and stage IV T-LBL in a global test on all proteins, a significant result was not reached. However, AKT S473/tot, S6RP S235/236 showed a stronger positive association with stage IV T-LBL (non-corrected p-values p=0.016 and p=0.029 from single tests), and LKB1 S428/tot with stage III T-LBL (non-corrected p-values p=0.070), as compared to the other proteins (**Figure 3A**). These three most differentially expressed proteins between stage IV (brown) and stage III T-LBL (orange) belonged to the AKT/mTOR pathway (**Figure 3B**). Noteworthy, this result was consistent with the significantly positive enrichment for genes involved in PI3K-AKT-mTOR signaling in stage IV compared to stage III T-LBL samples from our previously published dataset (9) (**Supplementary Figure S2G****)** and with Western Blot results related to the AKT pathway activation in stage IV T-LBL compared to stage III and T-ALL samples **(** **Supplementary Figure S3****).**

Overall, these results suggest that the AKT/mTOR pathway is hyperactivated in stage IV T-LBL compared to both T-ALL and stage III T-LBL. Accordingly, we found that stage IV T-LBL is characterized by a significantly increased expression of AKT S473/tot compared to both stage III T-LBL and T-ALL (**Figure 3C** and **Supplementary Figure S3**). Noteworthy, none of T-LBL patients was mutated on *PIK3CA* hot-spot exons and only 2/22 were mutated on *PTEN* exon 7, thus demonstrating the importance of phosphoproteomics in unraveling pathway activations that genomics alone cannot predict.

## Discussion

Despite evidence accumulating on a distinct genomic and transcriptomic profile between pediatric T-ALL and T-LBL, the distinction between these clinical entities and their treatment is still arbitrarily based on the percentage of blasts in the BM (6).

In the present study, we investigated the phosphoproteomic profile of pediatric T-ALL and T-LBL tumor cells, revealing that these diseases are also characterized by a different phosphoproteomic profile. In particular, we found that the FAK/ERK1/2 and AKT/mTOR pathway are more active in T-LBL compared to T-ALL tumor cells. Of note, these two pathways can interact since FAK affects the mTOR pathway through PI3K-AKT signaling activation, as demonstrated in a recent study on a breast cancer model, in which FAK inhibition was shown to reduce mTOR activation (27). Moreover, an increased activation of FAK/ERK1/2 in T-LBL compared to T-ALL is in agreement with the fundamental role that FAK/ERK1/2 pathway plays in sustaining and regulating the growth, angiogenesis and vascular permeability of solid tumors (28). Conversely, the JAK/STAT signaling pathway was globally more activated in T-ALL compared with T-LBL. The role of JAK/STAT pathway in normal lymphoid precursor cell proliferation, survival and differentiation has been widely described (29), as well as its pivotal role in hematological malignancies (30). In pediatric T-ALL, activating mutations of *JAK1* and *JAK3* and other genes involved in JAK/STAT signaling, such as *EP300*, *STAT5B*, *IL7R* and *PTPN11*, have been associated with an increased JAK/STAT signaling (31, 32) and, in the presence of *IL7R* mutations, with an increased resistance to steroid therapy (33). Similarly, also in T-LBL pediatric patients the hyperactivation of JAK/STAT pathway has been associated with the presence of TEL-JAK2 activating translocation and *JAK2* missense mutations (34). The reason why the JAK/STAT pathway is generally more active in T-ALL compared to T-LBL remains to be investigated.

Finally, with the aim of identifying a proteomic signature that can help in differentiating stage IV T-LBL from T-ALL patients, we successfully identified six proteins, namely ERK1/2 T202/Y204, AKT S473/TOT, mTOR S2448/tot, FAK Y397, P21 and BAX whose expression/activation was able to distinguish stage IV T-LBL from T-ALL. This finding, if confirmed in a larger validation cohort, could represent a diagnostic tool that can help discriminating between stage IV T-LBL and T-ALL. Intriguingly, AKT hyperphosphorylation alone was able to distinguish stage IV T-LBL from both T-ALL and stage III T-LBL.

Overall, these results could represent a starting point for the investigation of novel biomarkers that could discriminate stage IV T-LBL from T-ALL disease, so far based only on BM involvement criteria, paving the way for the identification of new therapeutic targets for highly aggressive stage IV T-LBL.

## Data Availability Statement

The original contributions presented in the study are included in the article/**Supplementary Material**. Further inquiries can be directed to the corresponding author.

## Ethics Statement

The studies involving human participants were reviewed and approved by Ethics Committee of Azienda Ospedaliera di Padova (protocol code 3623/AO/15, date of approval: 17/12/2015). Written informed consent to participate in this study was provided by the participants’ legal guardian/next of kin.

## Author Contributions

FL, VS and LM conceived the study; GV and FL selected and processed clinical samples for RPPA analysis; GV performed RPPA experiments; GC performed all the statistical analysis; GV and GC made the figures; GV, FL, VS and LM, interpreted data; EC and MaPi collected T-LBL clinical data; SC, MaPi, MaPr, SB, VC, AB and BB provided patients clinical care and biological samples; GV, FL, GC, VS and LM wrote the manuscript. BB and VC revised the manuscript. VS and LM supervised the experimental work and revised the manuscript. All authors approved the final version of the manuscript.

## Funding

This research has been funded by Associazione Italiana per la Ricerca sul Cancro, Milano, Italy (Investigator Grant – IG 2018 #21385 to LM and My First AIRC Grant – MFAG 2018 #21771 to VS) and Fondazione Città della Speranza (grant 21/03 to LM). The author GC was funded by grant PRIN2017 (20178S4EK9).

## Conflict of Interest

The authors declare that the research was conducted in the absence of any commercial or financial relationships that could be construed as a potential conflict of interest.

The reviewer LM declared a shared affiliation with the author VC to the handling editor at the time of review.

## Publisher’s Note

All claims expressed in this article are solely those of the authors and do not necessarily represent those of their affiliated organizations, or those of the publisher, the editors and the reviewers. Any product that may be evaluated in this article, or claim that may be made by its manufacturer, is not guaranteed or endorsed by the publisher.
