# Supplementary material for: Phosphoproteomic Analysis Reveals a Different Proteomic Profile in Pediatric Patients With T-Cell Lymphoblastic Lymphoma or T-Cell Acute Lymphoblastic Leukemia
Source: Front Oncol. 2022 Jul 8;12:913487. doi: 10.3389/fonc.2022.913487 (PMC9304622; doi:10.3389/fonc.2022.913487)
Supplement: Supplementary file 1 [file DataSheet_1.docx]

***Supplementary Material***

Supplementary Figures and Tables

**Table S1:** List of primary antibodies selected for RPPA staining. Each antibody was previously validated for single band specificity by Western Blot. Proteins analyzed in this study belong to the main deregulated pathways in leukemia and cancers, namely AKT/mTOR, JAK/STAT, RAS/MAPK, cell cycle and T-cell receptor signaling pathways.

| **Antibody** | **Company** | **Catalog number** |
| --- | --- | --- |
| 4EBP1 S65 | Cell Signaling Technology | 9456 |
| Acetyl-CoA Carboxylase 1S79 | Millipore | 04-1009 |
| AKT S473 | Cell Signaling Technology | 9271 |
| AKTT308 | Cell Signaling Technology | 13038 |
| AKT TOT | R&D | MAB1775 |
| AMPKα T172 | Cell Signaling Technology | 2535 |
| BAX | Cell Signaling Technology | 2772 |
| BCL-XL | Cell Signaling Technology | 2762 |
| BCL-2 S70 | Cell Signaling Technology | 2827 |
| B-RAF S445 | Cell Signaling Technology | 2696 |
| C-MYC | Millipore | OP10 |
| Cleaved CASPASE 7 (Asp198) | Cell Signaling Technology | 9491 |
| CDK2 | Cell Signaling Technology | 2546 |
| CK2α | Cell Signaling Technology | 2656 |
| CYCLIN B | BD bioscience | 610220 |
| CYCLIN E | BD bioscience | 51-1459GR |
| eIF4G S1108 | Cell Signaling Technology | 2441 |
| ERK1/2 T202/Y204 | Cell Signaling Technology | 9101 |
| FAK Y397 | BD bioscience | 611806 |
| GSK3α/β S21-9 | Cell Signaling Technology | 9331 |
| JAK1 Y1022/1023 | Cell Signaling Technology | 3331 |
| JAK2 Y1007/1008 | Cell Signaling Technology | 3771 |
| LCK TOT | Cell Signaling Technology | 2657 |
| LCK Y505 | Cell Signaling Technology | 2751 |
| LKB1 S428 | Cell Signaling Technology | 3051 |
| LKB1 TOT | Cell Signaling Technology | 3050 |
| MEK1/2 S217/221 | Cell Signaling Technology | 9154 |
| mTOR S2448 | Cell Signaling Technology | NBP1-51413 |
| mTOR TOT | NOVUSBIO | 4517 |
| p21 Waf1/Cip1 | Cell Signaling Technology | 2947 |
| p27 Kip1 | BD bioscience | 610241 |
| p38 T180/Y182 | Cell Signaling Technology | 4511 |
| p53 | Cell Signaling Technology | 9282 |
| p70 S6K T389 | Cell Signaling Technology | 9205 |
| PDK1 S241 | Cell Signaling Technology | 3061 |
| PKCα S657 | Millipore | 06822 |
| PKCΔ T505 | Cell Signaling Technology | 9374 |
| PKCθ T538 | Cell Signaling Technology | 9377 |
| PKCζ/λ T410/403 | Cell Signaling Technology | 9378 |
| PRAS40 T246 | BioSource | 44-1100 |
| PTEN S380 | Cell Signaling Technology | 9551 |
| PTEN TOT | Cell Signaling Technology | 9552 |
| RB S780 | BD bioscience | 558385 |
| RB TOT | BD bioscience | 554136 |
| S6RP S235/236 | Cell Signaling Technology | 2211 |
| SRC Y416 | Cell Signaling Technology | 2101 |
| SRC Y527 | Cell Signaling Technology | 2105 |
| STAT1 Y701 | Cell Signaling Technology | 9171 |
| STAT3 S 727 | Cell Signaling Technology | 9134 |
| STAT3 Y705 | Cell Signaling Technology | 9145 |
| STAT 5 Y694 | Cell Signaling Technology | 9351 |
| STAT6 Y641 | Millipore | 06937 |
| TYK2 Y1054/1055 | Cell Signaling Technology | 9321 |

**Figure S1:** Box-and-whisker plots of the 24 proteins that resulted differentially expressed between T-ALL and T-LBL pediatric patients, with a corrected FDR<0.05.


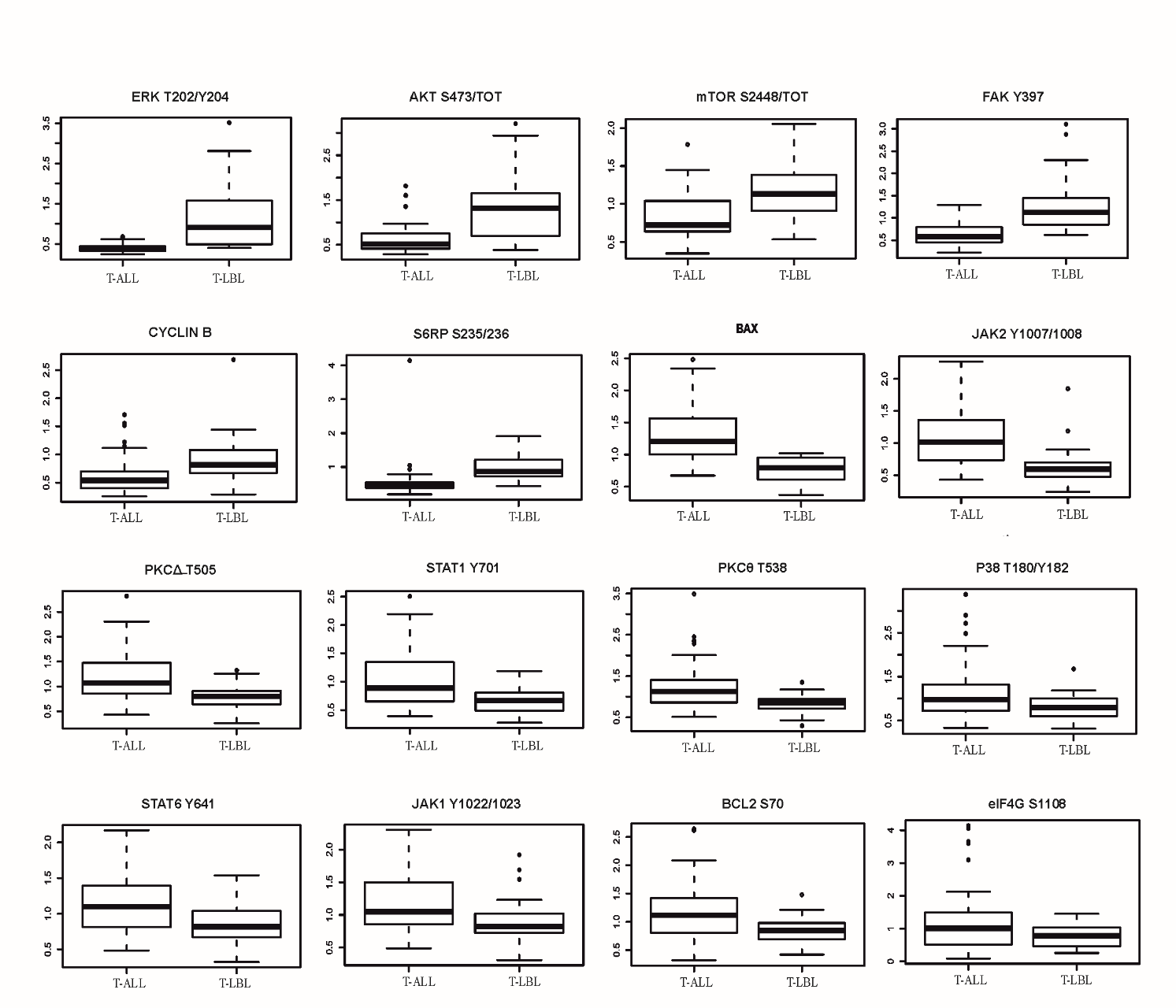

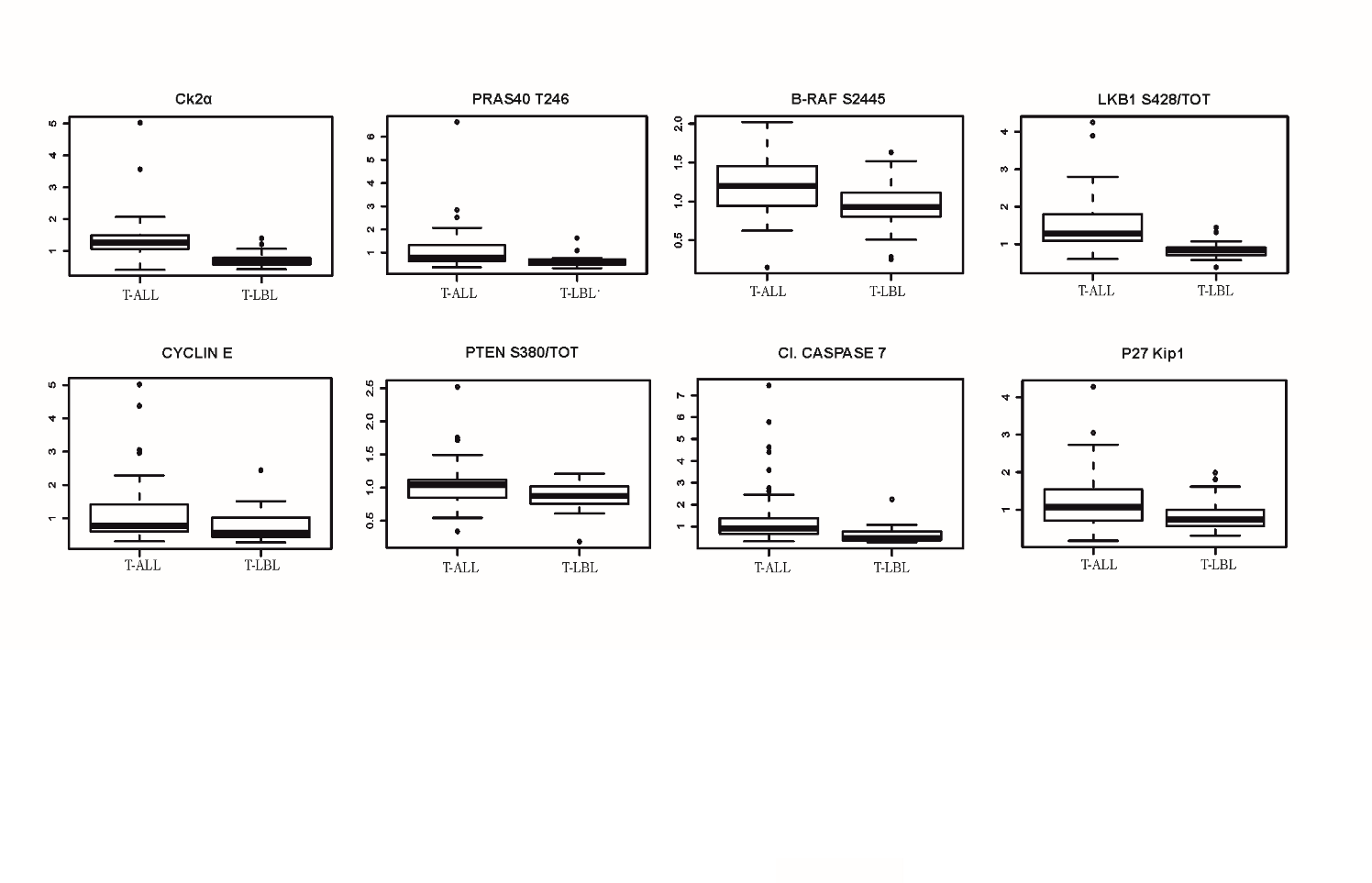


**Figure S2: Gene set enrichment analysis (GSEA) on gene expression data from Basso *et al.,* 2011.**

GSEA was performed using GSEA v4.2.3 with probe sets ranked by signal to noise and statistical significance determined by 1000 permutations. Gene set permutations were used to enable direct comparison between patients’ groups. Panels **A**, **B** and **C** show positive enrichment of the indicated genes set in T-LBL compared to T-ALL samples, panels **D**, **E** and **F** are referred to stage IV T-LBL compared to T-ALL, whereas panel G shows a significantly positive enrichment for genes involved in PI3K-AKT-mTOR signaling in stage IV compared to stage III T-LBL samples.

| **A** | **B** | **C** |
| --- | --- | --- |
| **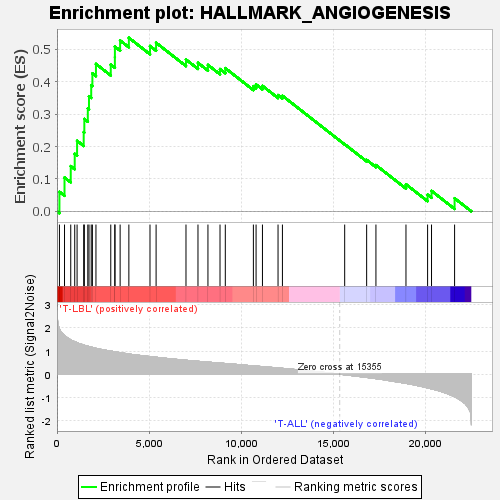** | **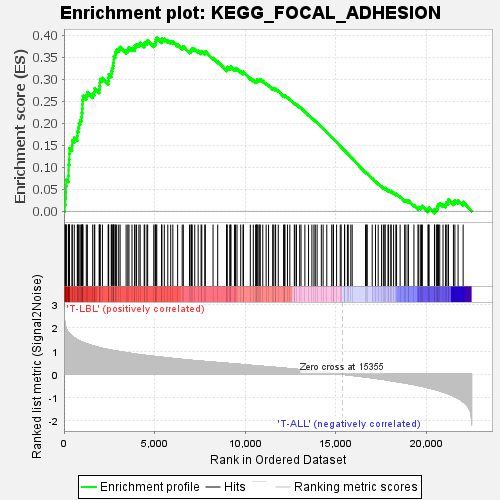** | **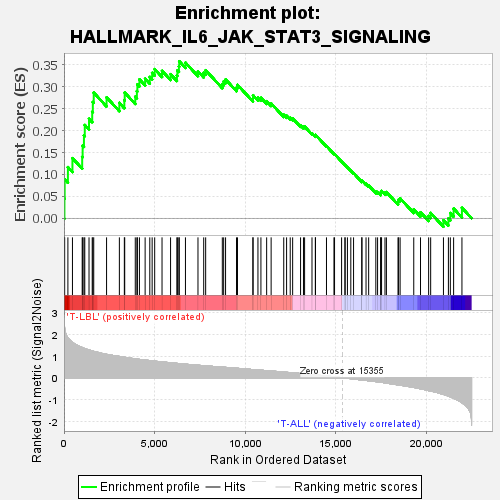** |
| **D** | **E** | **F** |
| **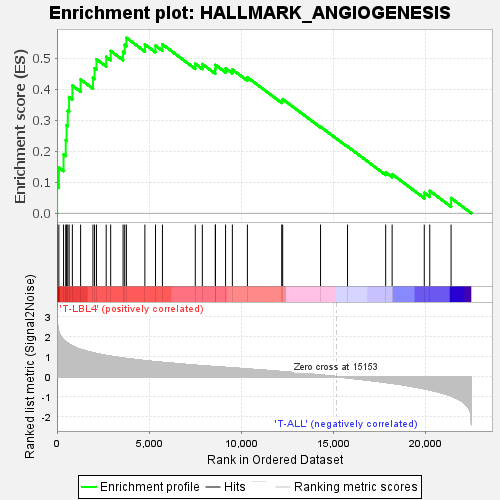** | **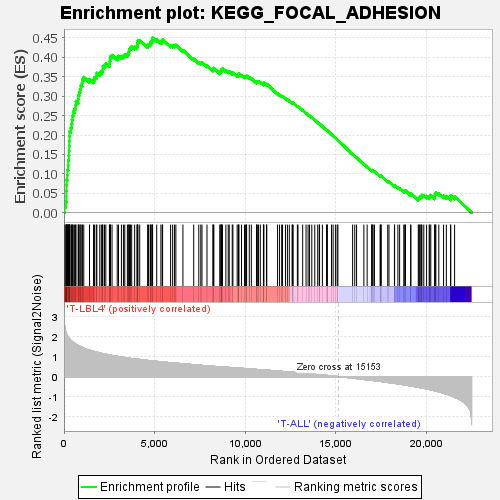** | **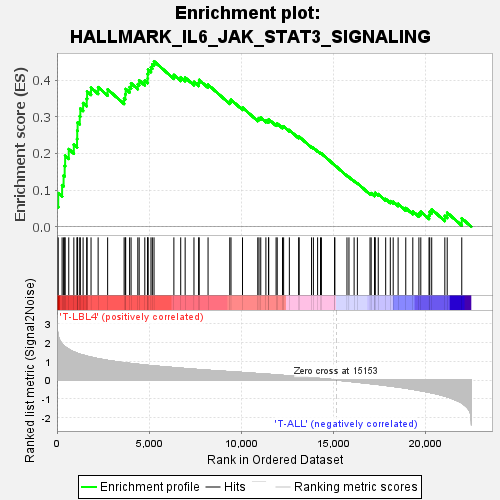** |
| **G** |  |  |
| **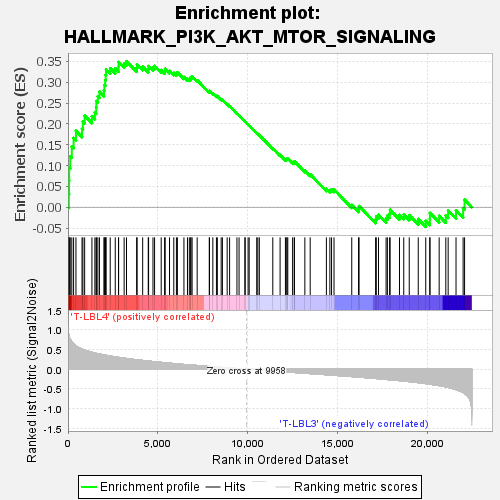** |  |  |

**Figure S3:** Western blotting results for activated AKT1 (AKT S473), total AKT1 (AKT TOT) and active S6 ribosomal protein phosphorylated at S235/236 (S6RP S235/236) in stage IV (n=5), stage III T-LBL (n=4) and T-ALL (n=5) samples at diagnosis.
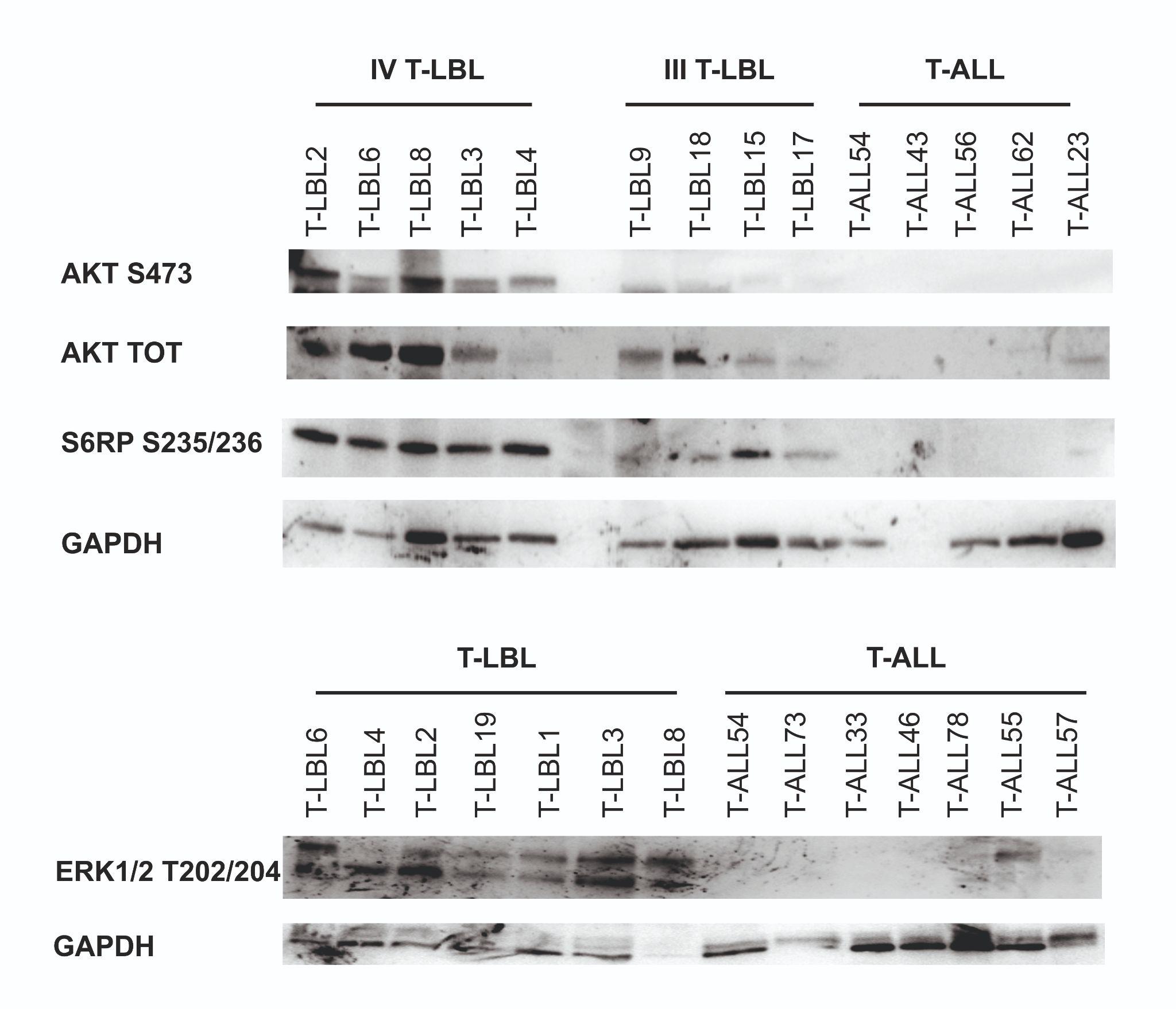


**Table S2:**  False discovery rate (FDR) values computed on p-values from the nonparametric Wilcoxon test between T-LBL and T-ALL pediatric patients. The FDR method (Benjamini-Hochberg procedure) was used for multiplicity corrections of statistical tests, and proteins with a result of FDR ≤ 0.05 were considered significantly differentially expressed/activated.

| **Protein** | **MEAN T-ALL** | **MEAN T-LBL** | **FDR (BH)** |
| --- | --- | --- | --- |
| BAX | 1.3111 | 0.7592 | 0.00000000 |
| LKB1 S428/tot | 1.5132 | 0.8368 | 0.00000027 |
| CK2𝛼 | 1.3822 | 0.7327 | 0.00000027 |
| PKC𝛥 T505 | 1.2225 | 0.7827 | 0.00005204 |
| PKC𝜃 T538 | 1.2573 | 0.8303 | 0.00008663 |
| STAT1 Y701 | 1.0449 | 0.6731 | 0.00015584 |
| JAK2 Y1007/1008 | 1.0977 | 0.6589 | 0.00017777 |
| FAK Y397 | 0.6300 | 1.3476 | 0.00062635 |
| S6RP S235/236 | 0.5433 | 0.9950 | 0.00080388 |
| AKT S473/tot | 0.6285 | 1.4385 | 0.00084434 |
| Cleaved CASP7 | 1.4090 | 0.6184 | 0.00094596 |
| ERK1/2 T202/Y204 | 0.3998 | 1.1728 | 0.00103667 |
| mTOR S2448/tot | 0.8158 | 1.1609 | 0.00192288 |
| BCL2 S70 | 1.1814 | 0.8719 | 0.00271168 |
| PRAS40 T246 | 1.0848 | 0.6424 | 0.00452343 |
| P38 T180/Y182 | 1.1725 | 0.8336 | 0.00839429 |
| PTEN S380/tot | 1.0418 | 0.8464 | 0.00873067 |
| STAT6 Y641 | 1.1470 | 0.8737 | 0.01016668 |
| BRAF S445 | 1.2166 | 0.9443 | 0.01123202 |
| EIF4G S1108 | 1.1607 | 0.7692 | 0.02489316 |
| CYCLIN B | 0.6187 | 0.9188 | 0.03015065 |
| P27^Kip1^ | 1.1910 | 0.8509 | 0.03262955 |
| JAK1 Y1022/1023 | 1.1537 | 0.9226 | 0.05651239 |
| CYCLIN E | 1.1298 | 0.7640 | 0.05661496 |
| STAT3 Y705 | 0.7093 | 0.9471 | 0.06163454 |
| P21 | 0.4855 | 0.8646 | 0.08125543 |
| STAT3 S727 | 0.8921 | 1.1097 | 0.09021990 |
| PKC𝛼 S657 | 1.2717 | 0.8761 | 0.11153664 |
| ACC S79 | 0.8495 | 1.0651 | 0.13374747 |
| 4EBP1 S65 | 1.0185 | 0.9484 | 0.15346046 |
| CDK2 | 0.8042 | 0.9854 | 0.24797111 |
| STAT5 Y694 | 1.0503 | 0.8406 | 0.28011187 |
| BCL-XL | 1.1534 | 1.0322 | 0.29670749 |
| GSK3𝛼/𝛽 S21 | 0.7723 | 0.9659 | 0.30195880 |
| TYK2 Y1054/1055 | 0.9428 | 0.8495 | 0.30195880 |
| RB S780/tot | 1.1851 | 1.8360 | 0.30195880 |
| LCK Y505/tot | 1.0606 | 1.4997 | 0.30195880 |
| C-MYC | 1.0618 | 0.9255 | 0.36757369 |
| P53 | 0.9939 | 1.0559 | 0.36757369 |
| P70 T389 | 0.9833 | 1.0809 | 0.41464417 |
| AMPK𝛼 T172 | 1.2033 | 1.0505 | 0.44138031 |
| SRC Y416/tot | 1.1229 | 0.9482 | 0.46760477 |
| PKC𝜁/𝜆 T410/403 | 1.1807 | 1.1098 | 0.53325088 |
| MEK S217/221 | 1.0477 | 1.0908 | 0.56811173 |
| PDK1 S241 | 1.1304 | 1.0984 | 0.72120097 |
| AKT T308/tot | 1.0978 | 1.0733 | 0.79663649 |
| SRC Y527/tot | 0.9230 | 0.9636 | 0.81562343 |

**Table S3:** List of *p-values* derived from a locally most powerful test (Global Test) [(Goeman et al., 2006)](https://paperpile.com/c/eV9lzu/jGPn), related to pathways globally evaluated for all considered proteins. Correction for multiple testing was performed by using the FDR method (Benjamini-Hochberg procedure).

| **Pathways** | **FDR (BH)** |
| --- | --- |
| **TCR pathway:** LCK Y505/tot; SRC Y527/tot; SRC Y416/tot | 0.329 |
| **mTOR/AKT pathway:** 4EBP1 S65; GSK3α/𝛽 S21; mTOR S2448/tot; PTEN S380/tot; PDK1 S241; S6RP S235/236; PRAS40 T246; AKT S473/tot; AKT T308/tot; P38 T180/Y182; P70 T389; EIF4G S1108; CK2𝛼 | <0.001 |
| **AMPK pathway:** AMPKα T172; LKB1 S428/tot; ACC S79 | 0.001 |
| **Cell cycle pathway:** P27^kip1^; RB S780/tot; CDK2; CYCLIN E; CYCLIN B; P21; P53; C-MYC | 0.002 |
| **JAK/STAT pathway:** STAT3 S727; STAT3 Y705; STAT1 Y701; JAK1 Y1022/1023; JAK2 Y1007/1008; STAT6 Y641; TYK2 Y1054/1055; STAT5 Y694 | 0.002 |
| **PKCs pathway:** PKCδ T505; PKC𝜃 T538; PKC𝜁/𝜆 T410/403; PKC𝛼 S657 | 0.004 |
| **FAK pathway:** FAK Y397; BRAF S445; ERK1/2 T202/Y204; MEK S217/221 | <0.001 |

**Table S4:**  FDR values computed on p-values from the nonparametric Wilcoxon test between IV T-LBL and T-ALL pediatric patients. The FDR method (Benjamini-Hochberg procedure) was used for multiplicity corrections of statistical tests, and only proteins with a result of FDR ≤ 0.05 were considered significantly differentially expressed/activated, and reported in the table.

| **Protein** | **MEAN T-ALL** | **MEAN IV T-LBL** | **FDR (BH)** |
| --- | --- | --- | --- |
| CK2𝛼 | 1.3822 | 0.6303 | 0.00056800 |
| S6RP S235/236 | 0.5433 | 1.2709 | 0.00056800 |
| ERK1/2 T202/Y204 | 0.3998 | 1.4875 | 0.00056800 |
| LKB1 S428/tot | 1.5132 | 0.7136 | 0.00056800 |
| BAX | 1.3111 | 0.7137 | 0.00074190 |
| FAK Y397 | 0.6300 | 1.7028 | 0.00074190 |
| JAK2_Y1007/1008 | 1.0977 | 0.5646 | 0.00244950 |
| AKT S473/tot | 0.6285 | 2.0744 | 0.00336130 |
| PRAS40 T246 | 1.0848 | 0.5595 | 0.00756180 |
| mTOR S2448/tot | 0.8158 | 1.2889 | 0.00869520 |
| P21 | 0.4855 | 0.8171 | 0.00869520 |
| PKC𝜃 T538 | 1.2573 | 0.7490 | 0.00959560 |
| PKC𝛥 T505 | 1.2225 | 0.7309 | 0.00959560 |
| JAK1 Y1022/1023 | 1.1537 | 0.7787 | 0.02901600 |
| STAT6 Y641 | 1.1470 | 0.7765 | 0.04012900 |
| CYCLIN B | 0.6187 | 0.8942 | 0.04012900 |
| STAT1 Y701 | 1.0449 | 0.6660 | 0.04968080 |
